# O.1.3-1 Senior stakeholder views and experience of cross-sector engagement and collaboration to improve long-term health and wellbeing in Wales

**DOI:** 10.1093/eurpub/ckad133.098

**Published:** 2023-09-11

**Authors:** Vasiliki Kolovou, Nicola Bolton, Diane Crone

**Affiliations:** Cardiff Metropolitan University, UK; kolovouvasiliki@outlook.com; Cardiff Metropolitan University, UK; kolovouvasiliki@outlook.com; Cardiff Metropolitan University, UK; kolovouvasiliki@outlook.com

## Abstract

**Purpose:**

We aimed to explore the challenges and opportunities to developing and implementing a strategic long-term national physical activity, health and wellbeing strategy. Wales has created innovative legislation through the Wellbeing of Future Generations (Wales) Act 2015 (WBFGA) and continues to drive progress on long-term strategies for health and wellbeing, with physical activity as a key outcome.

**Methods:**

Senior stakeholders (n = 11) that held leadership roles within the sport, environment, central/ local government, healthcare and public health participated in semi-structured, in-depth interviews (July - September 2022). The interviews were transcribed and analysed through Braun & Clarke’s (2014) thematic analysis using NVivo software.

**Results:**

The major themes related to: influencing within and outside the leaders’ own organisation; advocating long-term health strategies; protecting the allocated resources to long-term health outcomes from short-term pressures; and disruptive changes such as Covid-19 and government election cycles. The participants felt that physical activity is relevant to multiple strategies and departments within government, so resources should be “pooled together” across central government budgets to meet the needs of the populations and deliver on long-term strategies. Spanning sector and organisation boundaries was viewed as essential by all senior leaders and some noted that partnerships should be led with purpose and authenticity. The innovative WBFGA was mentioned as the key facilitator for creating shared accountability and a shared vision for Wales. The relatively small size of Wales was noted as an additional facilitator to forging positive and long-standing relationships with individuals and organisations. The participants recognised a key element of their leadership role which was engaging and collaborating with strong intent and authenticity.

**Conclusions:**

The value and role of spanning boundaries to promote and establish long-term strategies for physical activity, health and wellbeing remains largely uncaptured and not well understood. This study contributed to understanding the perspectives of senior leaders, from diverse sectors, on developing and implementing long-term health strategies. The contemporary shift towards whole systems approaches and long-term partnerships to deliver healthier future generations, will require better understanding of what works for engaging wider stakeholders and maintaining momentum to a shared agenda.

